# Cervical hibernoma and lipoblastomatosis

**DOI:** 10.1590/S1679-45082013000100020

**Published:** 2013

**Authors:** Carlos Eduardo Molinari Nardi, Leonardo Barreto, Leda Viegas de Carvalho, André Vicente Guimarães

**Affiliations:** 1 Núcleo de Cirurgia de Cabeça e Pescoço de Santos, Santos, SP, Brazil; 2 Hospital Ana Costa, Santos, SP, Brazil; 3 Hospital Irmandade da Santa Casa da Misericórdia de Santos, Santos, SP, Brazil

**Keywords:** Lipoma, Head and Neck Neoplasms, Biopsy, Biopsy, needle

## Abstract

Lipoblastoma and lipoblastomatosis are rare benign soft-tissue tumoral lesions resembling fetal adipose tissue. A total of 16 cases of lipoblastoma of the neck were reported in the literature, and only 3 were described in the posterior side of the neck. Hibernoma is a rare benign adipose tumor composed of brown fat cells and only about ten cases occurring in the cervical area have been reported. We reported two rare cases of adipose tissue tumors. The first case was a male infant aged 12 months who had a cervical mass on the posterior side of the neck. He underwent a complete resection of the lesion and the pathologic study revealed lipoblastomatosis. The second case was a 36-year-old man with an anterior cervical mass, which moved with swallowing. A resection was made and the histological analysis showed hibernoma.

## RESUMO

Lipoblastoma e lipoblastomatose são lesões tumorais benignas dos tecidos moles similares ao tecido adiposo fetal. Na literatura, apenas 16 casos de lipoblastoma na região do pescoço foram relatados e, destes, somente 3 foram descritos na região posterior do pescoço. Hibernomas são tumores benignos raros do tecido adiposo composto por células gordurosas marrons. Foram descritos cerca de dez casos desses tumores na região cervical. Este artigo relatou dois casos raros de tumores do tecido adiposo. No primeiro caso, tratouse de criança do gênero masculino, com 12 meses de idade, com aparecimento de massa cervical na região posterior do pescoço. A ressecção completa da lesão foi realizada, e o estudo patológico revelou lipoblastomatose. O segundo caso ocorreu em um homem de 36 anos com massa cervical anterior, que se movia com a deglutição. Após ressecção à análise histopatológica mostrou hibernoma.

## INTRODUCTION

Lipoblastomas and their diffuse, multicentric and infiltrative forms, the lipoblastomatosis, are rare benign soft tissue tumors that resemble the fetal adipose tissue. Adipose tumors comprise about 6% of soft tissue neoplasms and are developed in the first two decades of life. In general, 94% are lipomas, 4.7% lipoblastomas and 1.3% liposarcomas^(1)^. Lipoblastoma is a benign tumor of embryonic fat cells^(2)^ that occurs mainly before the age of 3 years, are more prevalent in men^(1)^, and arise primarily in the trunk and extremities. Only 16 cases of lipoblastoma in the neck have been reported in the literature^(2)^, and only 3 cases were described on the posterior side of the neck^(1)^. Cervical hibernoma is a benign tumor that could be considered a differential diagnosis and is opposed to lipoblastoma^(2)^, and occurs mainly in adults^(3)^. Only ten cases have been described in the neck^(4)^. The symptoms are: compression of cervical structures, respiratory compromise, Horner's syndrome and hemiparesis^(2)^.

We reported two cases of these rare lesions. The first one was a cervical hibernoma and the other a cervical lipoblastomatosis; both were found on the posterior neck.

## CASE REPORTS

### Case 1

**Figure 1:**
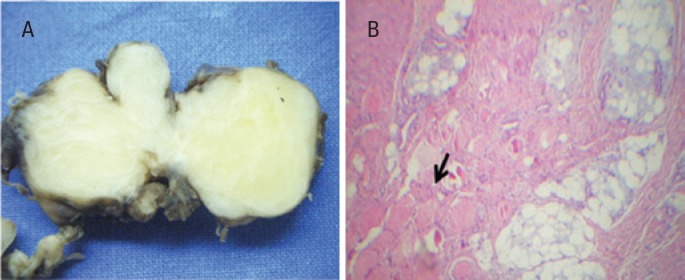
(A) Lipoblastomatosis – grossly, the tumor shows vague lobularity and fibrous areas. (B) Lipoblastomatosis showing muscle fibers entrapped into the lesion (arrow)

Male infant aged 12 months who had a cervical mass on the posterior side of the neck measuring 60×40mm. He underwent diagnostic imaging studies that showed a density fat tumor. After, a complete resection of the lesion was performed. The histological analysis showed an admixture of mature and immature adipocytes with muscle fibers entrapped and foci with a mixoyd stroma, which confirmed the presence of lipoblastomatosis (Figure 1).

### Case 2

A 36-year-old man with anterior cervical mass for 40 days that measured 50mm, had fibroelastic consistency and mobile with swallowing. Ultrasonography examination showed a thyroid nodule. The patient underwent a fine-needle aspiration. The cytological diagnosis was adipocytic tumor which confirmed the presence of hibernoma. After the complete resection, the histological exam confirmed the diagnosis of hibernoma.

## DISCUSSION

Adipose tumors comprise about 6% of soft tissue neoplasms and are developed in the first two decades of life. In general, 94% are lipomas, 4.7% lipoblastomas, and 1.3% liposarcomas. Cervical lipoblastomas are rare and constitute 10 to 15% of all lipoblastoma cases^(1)^. Hibernoma is a rare benign tumor of embryonal brown fat^(2)^, and only about ten cases of it are reported in the neck^(4)^.

Lipoblastoma are characterized as single subcutaneous nodule or by multiple lesions^(3)^. It grows rapidly and often is asymptomatic, except when causing mass effect^(1)^. On the other hand, hibernoma presents firm and mobile tumor, grows slowly and occurs chiefly in adults^(3)^; both hibernoma and lipoblastoma can cause mass effect^(5)^.

Diagnostic imaging studies are invaluable in the preliminary diagnosis of fatty tumors^(2)^. Plain X-rays and computed tomography scan can suggest the fat density of the tumor. Results of ultrasonography examinations can be confusing. The MRI is the most reliable method showing location, size, extent and mass characteristics of the tumor^(1)^, suggesting the histological components^(2)^.

The differential diagnosis of lipomatous tumors should include lipoma, lipoblastoma, hibernoma, and liposarcoma^(2)^. The lipoma is the most common soft tissue tumor and may show cytologic similarities to lipoblastoma. Although vacuoles may be seen in the adipocytes of conventional lipomas, the extent of vacuolated adipocytes is greater in number and smaller in lipoblastoma^(3)^. Hibernoma has been well cytologically described. Hibernoma cells differ from those of lipoblastoma, and they usually show a central nucleus and abundant finely-granular or microvacuolated cytoplasm. In addition, smears show no myxoid matrix and less capillaries^(3)^. Furthermore, hibernoma shows a lobular pattern, but it is entirely composed of brown fat with eosinophilic granular cytoplasm characteristic, that is not found in white fat of lipoblastoma^(2)^. Liposarcoma is histologically different from lipoblastoma. It is important to recognize that both tumors may contain mitotic figures, and also that lipoblastoma is a mass of fetal fat cells varying in different degrees. Despite both tumors could contain a plexiform capillary network, this feature tends to be more prominent in myxoid liposarcoma. It is particularly important to differentiate myxoid liposarcoma from lipoblastoma because both tumors have a myxoid background. The characteristic lobulation of lipoblastoma is a feature that is often absent in liposarcoma, but can be seen in the myxoid variant. Hyperchromasia and nuclear atypia are seen in liposarcoma, but absent in lipoblastoma, and may be the most distinguishing feature^(2)^.

Different lipomatous tumors tend to have characteristics of chromosomal abnormalities. Lipomas have abnormalities of chromosomes 12, 6, or 13, whereas myxoid liposarcoma tends to have the translocations t(12;16)(q13;p11) or t(12;22)(q13;q12). Hibernoma shows rearrangement in chromosome 11q13^2^ and lipoblastomas show rearrangement in chromosome 8q11-13.

Despite well-localized and nonmalignant nature of lipoblastomas, their rapidly growing might cause compressive symptoms. The standard therapy for cervical lipoblastoma is the complete surgical excision^(2)^. Its prognosis is good. By far the a recurrence rate of 15% has been reported for cervical lipoblastoma^(1)^. Lipoblastomatosis has a high propensity for recurrence^(2)^.

Curative treatment to hibernomas is the complete excision with preservation of vital structures^(4)^. This procedure must be made without technical difficulties since hibernoma usually presented as a well-circumscribed mass^(5)^. Unlike lipomas, the hibernomas has an extensive vascularity that should be carefully treated to avoid postoperative bleeding or hematoma. No case of recurrence or metastasis has been reported^(4,5)^. Additional treatment is not necessary, except when liposarcoma is suspected^(5)^.

## CONCLUSION

When dealing with a cervical mass, lipoblatomatosis and hibernoma should be considered in the differential diagnosis. The surgical treatment must be considered.
